# Isolation and Characterization of Yeasts Able to Assimilate Sugarcane Bagasse Hemicellulosic Hydrolysate and Produce Xylitol Associated with* Veturius transversus* (Passalidae, Coleoptera, and Insecta)

**DOI:** 10.1155/2017/5346741

**Published:** 2017-06-06

**Authors:** Italo Thiago Silveira Rocha Matos, Enedina Nogueira Assunção, Edson Junior do Carmo, Verena Makaren Soares, Spartaco Astolfi-Filho

**Affiliations:** Departamento de Genética, Universidade Federal do Amazonas, Av. Gal. Rodrigo Octávio, 3000 Manaus, AM, Brazil

## Abstract

Yeasts are an important component of insect gut microbial content, playing roles such as degradation of polymers and toxic compounds, biological control, and hormone, vitamin, and digestive enzyme production. The xylophagous beetle gut is a hyperdiverse habitat and a potential source of new species with industrial abilities such as enzyme production, pentose fermentation, and biodetoxification. In this work, samples of* Veturius transversus* (Passalidae, Coleoptera, and Insecta) were collected from the Central Amazon Rainforest. Their guts were dissected and a total of 20 microbial colonies were isolated using sugarcane bagasse hemicellulosic hydrolysate. They were identified as having 10 distinct biochemical profiles, and genetic analysis allowed identification as three clades in the genera* Candida*,* Williopsis,* and* Geotrichum*. All colonies were able to assimilate D-xylose and 18 were able to produce xylitol, especially a strain of* Geotrichum*, with a maximum yield of 0.502 g·g^−1^. These results agree with a previous prediction that the microbial community associated with xylophagous insects is a promising source of species of biotechnological interest.

## 1. Introduction

Yeasts are microorganisms of the Fungi kingdom, distributed in the phyla Ascomycota, Basidiomycota, and Deuteromycota [1]. On the other hand, beetles are the most abundant order of insects (Coleoptera, Insecta, Arthropoda, and Metazoa), with more than 400,000 species currently described [2].

The association of yeasts with beetles was decisive in the evolutionary success of these insects, the microbiota being indispensable to them, playing fundamental roles such as in the synthesis of amino acids, lipids, pheromones, and digestive enzymes and in biodetoxification [3, 4]. According to Suh et al. [5], the microbial content of a xylophagous beetle's gut is a hyperdiverse source of undescribed species.

Xylitol is a promising polyol of five carbons, with medical applications in middle ear otitis [6] and obesity prevention [7]. It is obtained from D-xylose reduction, performed by microbial fermentation or a chemical process [8]. As this latter releases a high number of by-products, demanding several steps for purification, sampling efforts aiming to isolate microbes with ability to produce xylitol keep being necessary.

Recent yeast and yeast-like fungi sampling efforts have increased the number of known species and strains able to produce vitamins, enzymes, and other products from fermentation of sugars such as ethanol and xylitol [9]. In this way, isolation and characterization of wild-type yeasts and yeast-like fungi remains an important approach.

The aim of this work was to isolate and characterize yeasts associated with the xylophagous beetle* Veturius transversus* (Passalidae, Coleoptera, and Insecta) able to assimilate sugarcane bagasse hemicellulosic hydrolysate (SBHH) as sole carbon source and produce xylitol by D-xylose fermentation.

## 2. Material and Methods

Under authorization (protocol number 34652-1) of the Instituto Chico Mendes de Conservação da Biodiversidade (Brazilian authority for biodiversity access), 15 beetles were collected from the Central Amazon Rainforest (3°06′05.20′′ S, 59°58′23.14′′ W). They were identified as* V. transversus*, a highly representative passalid beetle in this region. Three samples were deposited in the Entomological collection Paulo Burnheim (UFAM, Brazil).

The beetles were washed in 70% ethanol for 1 min, the elytra were removed, and the gut was dissected. Fragments of intestine of about 1 cm were incubated for 48 h (120 rpm, 28°C) in tubes with 10 mL of SBHH, prepared as previously described [10, 11]. After this time, 100 *µ*L of this suspension was spread on SBHH added to agar. Yeasts and yeast-like colonies were isolated in Petri dishes containing Sabouraud agar (yeast extract, 10 g/L; glucose, 40 g/L; agar, 20 g/L).

To evaluate their ability to ferment D-xylose, a loopful of each isolate was cultured in tubes containing 10 mL of YNBX medium (yeast nitrogen base without amino acids, 6.7 g/L; D-xylose, 40 g/L). After 7 days of incubation at 28°C and 120 rpm, the medium content was centrifuged and the supernatant was analysed by an HPLC system using a Rezex RPM monosaccharide column (300 × 7.8 mm, Pb^2+^ 8%, Phenomenex). The D-xylose consumption rate (%) was calculated according to the final and initial D-xylose concentrations. Xylitol yield (g·g^−1^) was calculated by the ratio xylitol produced* *:* *D-xylose consumed.

For taxonomic identification, biochemical characterization was performed using kit ID32C (BioMerieux®), according to the manufacturer's instructions. The results were plotted in the online application ApiWeb® (https://apiweb.biomerieux.com) for physiological similarity identification.

Furthermore, the isolates were evaluated by genomic internal transcribed spacer (ITS) and ribosomal gene nucleotide sequences. The DNA was extracted according to Harju et al. [12] and amplified by PCR using primers ITS1 (5′ TCC GTA GGT GAA CCT GCC 3′) and ITS4 (5′ TCC TCC GCT TAT TGA TAT GC 3′). The PCR products were used to perform a sequencing reaction using a BigDye® kit (Applied Biosystems), and nucleotide sequences were obtained in an Applied Biosystems 3130 Genetic Analyzer® automatic sequencer.

The obtained sequences were compared to the NCBI database (https://www.ncbi.nlm.nih.gov/) using BLAST (Basic Local Alignment Search Tool) and deposited in GenBank. For phylogenetic relationship analysis, nucleotide sequences were aligned using Clustal-W and analysed by neighbour-joining (bootstrap, 2000 replicas), provided by MEGA 6.0 [13]. Nucleotide sequences from the genomic ITS region of* Meyerozyma guilliermondii* (GenBank JN974905),* Trichosporon mycotoxinivorans* (GenBank JX891097), and* Scheffersomyces stipitis* (GenBank GU256745) were included in the phylogenetic tree as reference groups, this last being the external group.

## 3. Results

A total of 20 colonies were isolated and evaluated, correspondent to 10 species of four genera,* Candida* (12 isolates),* Cryptococcus* (five isolates),* Debaryomyces* (one isolate), and* Geotrichum* (two isolates); their biochemical profiles are described in Table 1.

Nucleotide sequence BLAST results identified three different groups, one close to* Candida tropicalis*, another composed of members of the species* Williopsis saturnus,* and the third composed of the genus* Geotrichum* sp. The fragment length was about 550 bp for* C. tropicalis* and* W. saturnus*, containing 18S rDNA (partial), ITS1 (complete), 5.8S rDNA (complete), ITS2 (complete), and 28S rDNA (partial). For* Geotrichum* sp., fragment length was on average 250 bp, containing ITS1 (partial), 5.8S rDNA (complete), and ITS2 (partial).

For isolates 07, 16, and 18, identity greater than or equal to 99% allows us to conclude that these are members of the species* W. saturnus*. Isolates 03, 08, 09, 10, 11, and 20 presented identity varying from 97% to 99% with* C. tropicalis*, making it admissible that they are closely related to this species, here named clade* C. tropicalis*. The other isolates presented identity varying from 92% to 96% with* Geotrichum* sp. or* Galactomyces* spp.

Considering the short length of the fragment, which allows classification only at genus level and the synonymy between* Geotrichum* and* Galactomyces*, they were classified as clade* Geotrichum* sp. The complete BLAST results and GenBank accession number of sequences are presented in Table 2.

**Figure 1:**
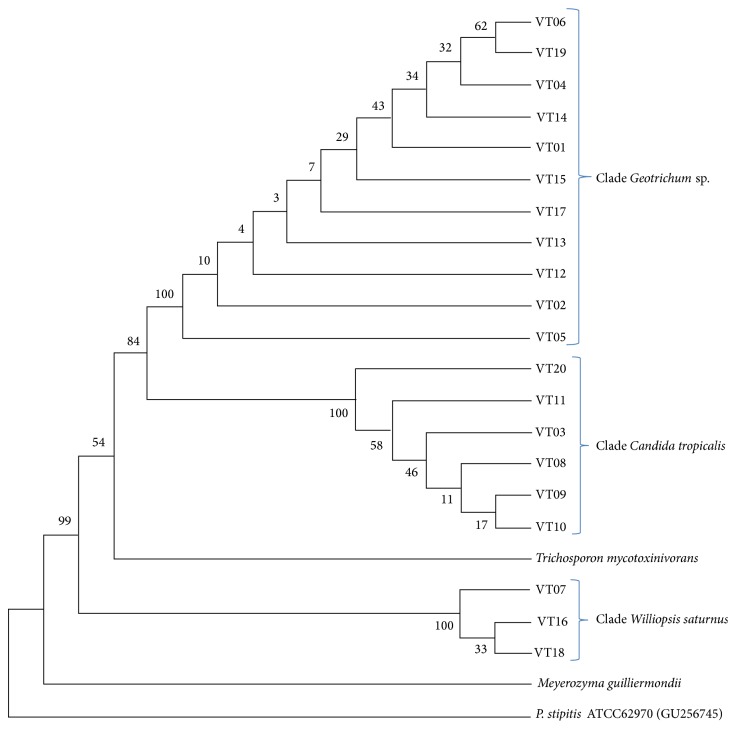
Neighbour-joining phylogenetic tree, endorsing the distribution of the isolates into three groups.

Neighbour-joining phylogenetic analysis endorsed the conclusion about the three groups that clade* W. saturnus* is close to* Meyerozyma guilliermondii* and* Scheffersomyces stipitis*, whereas* C. tropicalis* and* Geotrichum* sp. are closely related to* Trichosporon mycotoxinivorans*. The phylogenetic tree is presented in Figure 1.

Fermentation tests indicated that none of the isolates produces ethanol using xylose as carbon source. This result was expected because xylose fermentation to ethanol is an uncommon feature, being presented by less than 1% of known yeast species [14]. However, most of them were able to produce xylitol, only isolates 12 and 13 (*Geotrichum* sp.) being unable to do this. The highest yield was observed in isolate 01 (*Geotrichum* sp.), reaching 0.502 g·g^−1^ and consuming 92.6% of the D-xylose. The complete results are presented in Table 3.

## 4. Discussion

All isolates were able to assimilate D-xylose, a common feature in yeasts able to metabolize SBHH because this is the most abundant monosaccharide in hemicellulose [15].* Candida* is the most representative, but that occurs because there are a great number of asexual phase (anamorph) species classified in this genus, which is a polyphyletic group [5, 16].

The other genera,* Cryptococcus* and* Debaryomyces*, have remarkable biotechnological potential in incorporation of lipids in their biomass, being reported as oleaginous yeasts [17, 18]. Suh and Blackwell [19] describe the genus* Geotrichum* as dimorphic fungi, being anamorphs of the genera* Dipodascus* and* Galactomyces* and growing being yeast-like according to environmental conditions.

All biochemical profile results presented similarity with species able to perform pentose fermentation and/or another process with biotechnological potential. However, according to Barnett [20], biochemical profiles may be used as complementary information but cannot be conclusive for taxonomic identification because they can present high variation, with it being recommended to evaluate genomics data. According to Hou-Rui et al. [21], up to 1% of nucleotide substitution in a ribosomal domain is permitted for strains of a single biological species, rDNA sequence analysis being a simple and reliable tool for taxonomic identification.

The phylogenetic analysis endorses that predicted by Barnett [20], noticeable because clade* W. saturnus* is composed of isolates with three different biochemical profiles, whereas clade* C. tropicalis* is composed of five different biochemical profiles (one of those* C. tropicalis*) and clade* Geotrichum* has eight different biochemical profiles. Furthermore, there were some isolates with the same biochemical profile distributed in all clades, strengthening that hypothesis.

The maximum theoretical yield for xylitol production from D-xylose fermentation is 1.0 g·g^−1^. Despite this, as microbes produce xylitol as a compatible solute, it is excreted in osmotic stress conditions and then consumed as the medium becomes less harsh [22]; common yields range from 40% to 70% [23]. The highest yield value for microbial fermentation is reported by Granström et al. [24] for* Candida* sp., at 0.85 g·g^−1^.

With* Geotrichum* sp. (isolate 01) being a wild-type strain with the capability to produce xylitol like some industrial strains, it can be considered a promising xylitol-producing yeast. This is the first work to report xylitol production by wild-type yeast strains associated with beetles from the Central Amazon Rainforest.

## 5. Conclusion

The yeast community associated with* V. transversus* gut is rich in D-xylose-assimilating and xylitol-producing species, some of which present potential close to industrial strains.* Geotrichum* is a highly representative group in this community.

*Geotrichum* sp. (isolate 01) presents high xylitol yield, reaching about 50% of the maximum theoretical yield, and is a promising xylitol-producing strain.

Subsequent efforts must be concentrated on developing bioprocesses using these isolates.

## Figures and Tables

**Table 1:** Biochemical profiles of isolates from *V. transversus*, identified according to ApiWeb (BioMerieux).

Isolate	GAL	ACT	SAC	NAG	LAT	ARA	CEL	RAF	MAL	TER	2KG	MDG	SOR	XYL	RIB	GLY	RHA	PLE	ERY	MEL	GRT	MLZ	GNT	LVT	MAN	LAC	INO	GLU	SBE	GLN	ESC	Species	Similarity (%)
01	+	+	+	+	+	+	+	+	+	+	+	+	+	+	+	+	+	+	+	+	+	+	+	+	+	−	−	+	−	+	+	*Cryptococcus humicola*	98.4%
02	+	−	+	+	+	+	+	+	+	+	+	+	+	+	+	+	+	+	−	−	−	−	−	−	+	+	+	+	−	−	−	*Cryptococcus curvatus*	—
03	+	−	+	+	−	−	−	−	+	+	+	+	+	+	−	+	−	+	−	−	−	+	−	−	+	−	−	+	−	−	−	*Debaryomyces etchellsii*	79.1%
04	+	−	+	+	+	+	+	+	+	+	+	+	+	+	+	+	+	+	+	+	−	−	+	−	+	−	−	+	−	−	−	*Candida membranifaciens*	—
05	+	−	+	+	−	−	+	−	+	+	+	−	+	+	+	−	+	+	−	−	+	+	+	−	−	−	−	+	−	+	−	*Candida intermedia*	—
06	+	+	+	+	−	−	−	−	+	+	+	+	+	+	−	+	−	+	−	−	−	+	−	+	+	+	+	+	+	+	−	*Candida parapsilosis*	—
07	+	−	+	+	+	+	+	+	+	+	+	+	+	+	+	+	+	+	+	+	+	+	+	+	+	+	+	+	+	+	+	*Cryptococcus humicola*	99.2%
08	+	−	+	+	+	+	+	+	+	+	+	+	+	+	+	+	+	+	+	+	+	+	+	+	+	+	+	+	−	+	−	*Cryptococcus humicola*	99.7%
09	+	−	+	+	+	+	+	+	+	+	+	+	+	+	+	−	−	+	−	−	−	+	+	+	+	−	−	+	−	+	−	*Candida tropicalis*	53.6%
10	+	−	+	+	+	+	+	+	+	+	+	+	+	+	+	+	+	+	+	+	+	+	+	+	+	+	−	+	+	+	−	*Cryptococcus humicola*	99.5%
11	+	+	+	+	+	+	−	−	+	+	+	−	+	+	−	+	−	+	+	−	−	+	−	−	+	−	−	+	−	−	+	*Candida famata*	—
12	−	+	−	−	−	−	−	−	−	−	−	−	−	+	−	+	−	−	−	−	−	−	−	−	−	−	−	+	−	−	−	*Geotrichum capitatum*	97.7%
13	−	+	−	−	−	−	−	−	−	−	−	−	−	+	−	+	−	−	−	−	−	−	−	−	−	−	−	+	−	−	+	*Geotrichum capitatum*	97.7%
14	+	+	+	+	+	−	−	−	+	+	+	+	+	+	−	+	−	+	−	−	−	+	−	−	+	−	−	+	−	−	+	*Candida sake*	99.5%
15	+	+	+	+	+	−	−	+	+	+	+	−	+	+	−	+	−	+	−	−	−	+	−	−	−	−	−	+	+	+	−	*Candida sake*	99.0%
16	+	+	+	+	+	+	−	−	+	+	+	+	+	+	−	+	−	+	−	−	−	+	+	−	+	−	−	+	−	−	+	*Candida parapsilosis*	83.1%
17	+	+	+	+	+	−	+	−	+	+	+	+	+	+	+	+	−	+	−	−	−	+	−	−	+	−	+	+	−	+	−	*Candida tropicalis*	—
18	+	+	+	+	−	−	−	−	+	+	+	+	+	+	−	+	−	+	−	−	−	+	−	+	+	+	+	+	+	+	−	*Candida sake*	95.4%
19	+	+	+	+	+	−	−	−	+	+	+	+	+	+	−	+	−	+	−	−	−	+	−	−	+	+	−	+	−	+	+	*Candida tropicalis*	94.4%
20	+	−	+	+	−	+	+	−	+	+	+	−	+	+	+	−	−	+	−	−	+	+	+	−	+	−	−	+	+	+	+	*Candida intermedia*	95.7%

GAL: galactose, ACT: cycloheximide, SAC: sucrose, NAG: N-acetyl glucosamine, LAT: lactic acid, ARA: arabinose, CEL: cellobiose, RAF: raffinose, MAL: maltose, TRE: trehalose, 2KG: 2 keto-gluconate, MDG: *α*-methyl-glucopyranoside, SOR: sorbitol, XYL: xylose, RIB: ribose, GLY: glycerol, RHA: rhamnose, PLE: palatinose, ERY: erythritol, MEL: melibiose, GRT: sodium glucuronate, MLZ: melezitose, GNT: potassium gluconate, LVT: levulinic acid, MAN: mannitol, LAC: lactose, INO: inositol, GLU: glucose, SBE: sorbose, GLN: glucosamine, ESC: esculin iron citrate.

**Table 2:** Identification of isolates according to BLAST result and nucleotide sequence GenBank accession number.

Isolate	Species	Max identity (%)	Query coverage (%)	*e*-value	GenBank accession number
01	*Geotrichum* sp.	93	92	1*e* − 119	KP276644
02	*Geotrichum* sp.	95	93	3*e* − 131	KP276636
03	*Candida tropicalis*	99	98	0.0	KP276645
04	*Geotrichum* sp.	94	100	1*e* − 121	KP276637
05	*Galactomyces candidum*	96	97	1*e* − 131	KP276638
06	*Geotrichum* sp.	96	99	1*e* − 126	KP276639
07	*Williopsis saturnus*	99	100	0.0	KP257575
08	*Candida tropicalis*	98	99	0.0	KP276646
09	*Candida tropicalis*	99	99	0.0	KP276647
10	*Candida tropicalis*	98	99	0.0	KP276648
11	*Candida tropicalis*	98	99	0.0	KP276649
12	*Galactomyces candidum*	96	98	1*e* − 136	KP276640
13	*Galactomyces geotrichum*	96	98	1*e* − 136	KP276641
14	*Geotrichum* sp.	95	100	1*e* − 125	KP276642
15	*Geotrichum* sp.	94	100	3*e* − 102	KP288488
16	*Williopsis saturnus*	99	100	0.0	KP257574
17	*Geotrichum* sp.	93	99	7*e* − 129	KP288487
18	*Williopsis saturnus*	99	100	0.0	KP257573
19	*Geotrichum* sp.	94	99	5*e* − 134	KP276643
20	*Candida tropicalis*	97	99	0.0	KP276650

**Table 3:** D-xylose consumption rate (%) and xylitol yield of each isolate.

Isolate	D-xylose consumption rate (%)	Xylitol yield (g·g^−1^)
01	92.6	0.502
02	29.9	0.210
03	33.4	0.214
04	36.2	0.255
05	30.7	0.186
06	35.6	0.224
07	31.0	0.169
08	33.2	0.180
09	34.0	0.170
10	30.7	0.100
11	100.0	0.339
12	27.5	0.0
13	18.7	0.0
14	100.0	0.315
15	100.0	0.326
16	100.0	0.304
17	41.4	0.304
18	100.0	0.341
19	100.0	0.370
20	100.0	0.347
